# RTEF-1 Inhibits Vascular Smooth Muscle Cell Calcification through Regulating Wnt/β-Catenin Signaling Pathway

**DOI:** 10.1007/s00223-021-00833-4

**Published:** 2021-03-13

**Authors:** Jingjing Cong, Bei Cheng, Jinyu Liu, Ping He

**Affiliations:** 1 Department of Geriatrics, Union Hospital, Tongji Medical College, Huazhong University of Science and Technology, 1277 Jiefang Avenue, Wuhan, 430022 Hubei China; 2 Department of Rehabilitative Medicine, Wuhan NO.1 Hospital, Wuhan, 430022 Hubei Province China

**Keywords:** Vascular calcification, Related transcriptional enhancer factor, Vascular smooth muscle cells, Wnt, Β-catenin

## Abstract

Vascular calcification (VC) is highly prevailing in cardiovascular disease, diabetes mellitus, and chronic kidney disease and, when present, is associated with cardiovascular events and mortality. The osteogenic differentiation of vascular smooth muscle cells (VSMCs) is regarded as the foundation for mediating VC. Related transcriptional enhancer factor (RTEF-1), also named as transcriptional enhanced associate domain (TEAD) 4 or transcriptional enhancer factor-3 (TEF-3), is a nuclear transcriptional factor with a potent effect on cardiovascular diseases, apart from its oncogenic role in the canonical Hippo pathway. However, the role and mechanism of RTEF-1 in VC, particularly in calcification of VSMCs, are poorly understood. Our results showed that RTEF-1 was reduced in calcified VSMCs. RTEF-1 significantly ameliorated β-glycerophosphate (β-GP)-induced VSMCs calcification, as detected by alizarin red staining and calcium content assay. Also, RTEF-1 reduced alkaline phosphatase (ALP) activity and decreased expressions of osteoblast markers such as Osteocalcin and Runt-related transcription factor-2 (Runx2), but increased expression of contractile protein, including SM α-actin (α-SMA). Additionally, RTEF-1 inhibited β-GP-activated Wnt/β-catenin pathway which plays a critical role in calcification and osteogenic differentiation of VSMCs. Specifically, RTEF-1 reduced the levels of Wnt3a, p-β-catenin (Ser675), glycogen synthase kinase-3β (GSK-3β), and p-GSK-3β (Ser9), but increased the levels of p-β-catenin (Ser33/37). Also, RTEF-1 increased the ratio of p-β-catenin (Ser33/37) to β-catenin proteins and decreased the ratio of p-GSK-3β (Ser9) to GSK-3β protein. LiCl, a Wnt/β-catenin signaling activator, was observed to reverse the protective effect of RTEF-1 overexpression on VSMCs calcification induced by β-GP. Accordingly, Dickkopf-1 (Dkk1), a Wnt antagonist, attenuated the role of RTEF-1 deficiency in β-GP-induced VSMCs calcification. Taken together, we concluded that RTEF-1 ameliorated β-GP-induced calcification and osteoblastic differentiation of VSMCs by inhibiting Wnt/β-catenin signaling pathway.

## Introduction

Vascular calcification (VC) is a highly prevalent pathophysiological process that has been associated with aging, atherothrombotic cardiovascular disease, diabetes mellitus, and chronic kidney disease. When present, it portends a worse clinical outcome and predicts major adverse cardiovascular events as shown in several population-based studies [1]. However, no ideal approaches exist to prevent or reverse VC, partly because the mechanisms are heterogeneous and complex. VC, previously considered a passive degenerative condition of aging, is currently characterized as an active biomineralization process, with complicated signaling pathways, similar to osteogenesis [2]. Vascular smooth muscle cells (VSMCs) are of mesenchymal origin and, under stress, can differentiate into different mesenchymal-derived cell types, such as osteoblasts and chondrocytes, leading to calcification [3]. In calcified blood vessels, VSMCs undergo an osteogenic phenotypic change and upregulate the expression of mineralization-regulating proteins, thus contributing to VC [3, 4].

Related transcriptional enhancer factor (RTEF-1), also named as transcriptional enhanced associate domain (TEAD) 4 or transcriptional enhancer factor-3 (TEF-3), is a key member of the TEAD family. TEAD transcription factors are downstream effectors of the Hippo pathway, and they regulate cell proliferation, tissue regeneration, and metastasis [5]. The other three members of the TEAD family, such as TEAD1, TEAD2, and TEAD3, have the same domain structure: a DNA-binding TEA/ATTS domain in the N-terminus, but show tissue-specific expression, indicating that they have both common functions (regulating cell proliferation and contact inhibition) and distinct functions (heart development, neural development, and trophectoderm lineage determination) [6]. RTEF-1 is highly expressed in skeletal muscle; initial studies focused on its role in blastocyst formation and reported that TEAD4 is required for specification of the trophectoderm lineage in preimplantation embryos [7, 8]. Recently, RTEF-1 was demonstrated to be a novel prognostic marker of gastric cancer, breast cancer, colorectal cancer, melanoma, laryngeal cancer, and head-neck squamous cell carcinoma [6, 9]. Apart from its oncogenic role in the canonical Hippo-YAP/TAZ pathway, other studies have reported its participation in many pathological process of cardiovascular disorders, such as increasing endothelial-dependent microvascular relaxation [10], improving glucose clearance and insulin sensitivity in the endothelium [11], and stimulating cardiac hypertrophy in the myocardium [12], as well as accelerating recovery from ischemia [13]. However, the role and mechanism of RTEF-1 in VC, particularly in calcification of VSMCs, are poorly understood.

Wnts are secreted lipid-modified glycoproteins that activate cell surface receptor-mediated signal transduction pathways to regulate a variety of cellular activities, such as development, proliferation, migration, polarity, and differentiation [14]. The canonical Wnt/β‐catenin signaling pathway is the most studied Wnt pathway and plays an essential role in VC and can induce trans-differentiation of VSMCs to an osteogenic phenotype [15]. Under pro-calcifying conditions, the canonical Wnt/β‐catenin signaling pathway affects the osteogenic differentiation of VSMCs by regulating β-catenin levels and subcellular localization [16, 17]. Wnts initiate intracellular accumulation of β-catenin by binding to cell surface receptor complexes and then inhibiting glycogen synthase kinase-3β (GSK-3β)-mediated phosphorylation of β-catenin on Ser33/Ser37/Thr41 sites, thereby promoting β-catenin to enter into the nucleus where they affect gene expression. Besides the sites phosphorylated by GSK-3β, β‐catenin can be phosphorylated by protein kinase A (PKA) at the Ser675 site, which promotes the transcriptional activity of β-catenin [18]. Two forms of active β-catenin, dephosphorylated on Ser37/Thr41 and phosphorylated on Ser675 site, were upregulated by high phosphate in VSMCs [17]. Activation of Wnt/β-catenin signaling by high phosphate leads to Runt-related transcription factor-2 (Runx2) induction, which in turn, regulates pivotal processes essential for osteoblast differentiation and phenotypic characterization through governing the expression of bone-related proteins [19]. YAP/TAZ has been demonstrated to suppress the osteogenic differentiation of osteoblast progenitors by negatively regulating the Wnt/β‐catenin signaling and Runx2 activity during the embryonic development [20]. Whether the mechanism of RTEF-1 in calcification of VSMCs is associated with the Wnt/β‐catenin signaling remains unknown.

In this study, we used β-glycerophosphate (β-GP) induced in vitro model of VC to determine the effect of RTEF-1 in VSMCs calcification and osteogenic differentiation. Also, specific Wnt agonist and antagonist were used to explore whether the underlying mechanisms which may be involved in RTEF-1-driven anti-calcifying effect in VSMCs is mediating by inhibition of Wnt/β‐catenin signaling.

## Materials and Methods

### Cells Culture and Treatment

Human vascular smooth muscle cells (VSMCs) were purchased from ATCC and cells were cultured in Dulbecco's Modification of Eagle’s Medium (DMEM) containing 10% fetal bovine serum, 100 U/mL penicillin, and 100 U/mL streptomycin in a humidified 5% CO_2_ incubator at 37 °C. Cells were used between passages 3 and 6 for all experiments and the medium was changed every other day.

After reaching 80% confluence, VSMCs were incubated for 12 days in medium supplemented with 10 mM β-GP (G9422, Sigma, Silicon Valley, USA). For some experiments, VSMCs calcification was induced with 10 mM β‐GP after knockdown of RTEF-1 by small interfering RNA (siRNA) transfection or overexpression of RTEF-1 by stable trans-infectants. For manipulation, the activity of WNT/β-catenin signaling, LiCl (5 mmol/L), or Dickkopf-1 (Dkk1) (100 ng/ml) was added to the medium, as indicated in figure legends.

### Liposomal Transduction, Stable Cell Line Generation, and Small Interfering RNA Transfection

The coding sequence of RTEF-1 (NM_003213) is obtained from a pXJ40/RTEF-1 construct (gifted from Dr. Alexandre Stewart, University of Ottawa). The liposomal medium was used to transduce VSMCs, and stable trans-infectants were selected with G418 (500 lg/mL). Small interfering RNA (siRNA) encoding human RTEF-1 (Thermo Fisher Scientific, Beijing, China) at a final concentration of 50 μM was transfected using Lipofectamine 2000 (Invitrogen, Carlsbad, CA) and confirmed by real-time PCR and Western blot. Two duplexes of RTEF-1 siRNA were 5′-GGGCAGACCUCAACA CCAATT-3′, 5′-UUGGUGUUGAGGUCUGCCCAG-3′, and 5′-ACCCAAGAUGC UG.

UGUAU UTT-3′, 5′-AAUACACAGCAUCUUGGGUTT-3′. A duplex of RNA (5′-UU CUCCGAACGUGUCACGUTT-3′, 5′-ACGUGACACGUUCGGAGAATT-3′) that is not targeted to any human gene was used as a negative control.

### Calcification Assay

To induce calcification, human VSMCs were cultured in DMEM, supplemented with 10 mM β-GP for 12 d. Alizarin red S staining was used to examine the calcium deposition in cultured VSMCs. Briefly, cells were fixed in 4% formaldehyde for 10 min at room temperature, followed by incubation with 2% alizarin red (pH 4.2) for 30 min. After cells were washed with deionized water to remove non-specific staining, images were taken under an inverted phase-contrast microscope. Positively stained cells or calcified nodules showed a reddish/purple color. The calcium content in the HCl supernatant was subjected to colorimetric analysis using a Calcium Assay Kit (TC1026, Leagene, Beijing, China). Cells were washed with PBS and decalcified with 0.6 mol/L HCl for 24 h. Calcium content was normalized to the protein content. ALP activity was measured in a colorimetric analysis using an alkaline phosphatase (ALP) assay kit (E-BCK091, Elabscence, Wuhan, China) according to the manufacturer’s instructions.

### Quantitative Real-Time PCR

Total RNA was isolated from cells using Trizol reagent (9767, Takara, Shiga, Japan), and cDNA synthesis was performed using the PrimeScript RT Reagent Kit (AK3920, Takara, Shiga, Japan) according to the manufacturer’s instructions. Quantitative RT-PCR was performed in duplicate with Power SYBR Green PCR Master Mix (Applied Biosystems, ABI) according to the manufacturer’s protocol on an ABI 7500 sequence detection system. The sequences of the primer pairs used for PCR amplification were as follows: GAPDH, 5′-TCAAGAAGGTGGTGAAGCAGG-3′ (forward) and 5′-TCAAAGGTGGAGGAGTGGGT-3′ (reverse); Osteocalcin, 5′-AGGGCAGCGAGG TAGTGA-3′ (forward) and 5′-CCTGAAAGCCGATGTGGT-3′ (reverse); Runx2, 5′-AGGGCAGCGAGGTAGTGA-3′ (forward) and 5′-CCTGA AAGCCGATGTGGT-3′ (reverse); α-SMA (SM α-actin), 5′-GGCTATTCCTTCGTGACTACTG-3′ (forward) and 5′-AGCAG TGGCCATCTCATTT-3′ (reverse); Wnt3a, 5′-GCAGCAACAGTCTTACCT-3′ (forward) and 5′-ACAGGACTTGGGAGGTAT-3′ (reverse); GSK-3β, 5′-CTTACACC. CACCATCCCACT-3′ (forward) and 5′-CCTCCACAAA TTGCTGCTGT-3′ (reverse); β-catenin, 5′-GCCAAACCTAAGCACAAGC-3′ (forward) and 5′-GGAACAGGGAC. TCGCACT-3′ (reverse). The mRNA levels of target genes were calculated after normalization to GAPDH mRNA.

### Western Blot Analysis

Total protein was extracted from cells using a lysis buffer (50 mM Tris–HCl, pH 7.4, 150 mM NaCl, 1% Triton X-100, 1% sodium deoxycholate, 0.1% SDS, 1% PMSF). Cytosolic and nuclear proteins were separated using a NE-PER Nuclear and Cytoplasmic Extraction Reagents Kit (#26,616, Thermo Fisher Scientific) according to the manufacturer’s protocol. Protein concentration was measured using a BCA protein assay kit (P0009, Biyotime, Wuhan, China). Samples were mixed with an equal amount of 5X SDS loading buffer (125 mM Tris–HCl, 4% SDS, 20% glycerol, 100 mM DTT, and 0.2% bromophenol blue) and heated at 99 °C for 5 min. Subsequently, equal amounts of protein from each sample were separated by 8–12% SDS-PAGE gel and transferred onto a nitrocellulose membrane. Membranes were then blocked with 5% nonfat dry milk for 1 h at room temperature and incubated with primary antibody overnight at 4 °C, followed by incubation with appropriate secondary antibodies conjugated to Horseradish Peroxidase at room temperature for 60 min. The blotted membranes were visualized with an enhanced chemiluminescence kit in Image Lab system (Bio-Rad, USA). The protein levels of target genes were calculated after normalization to GAPDH mRNA. For quantification, relative protein expression was normalized to the GAPDH protein level. The primary antibodies were obtained from the following sources: anti-RTEF-1 (ab197589, Abcam, Cambridge, UK); anti-GAPDH (AF7021, Affinity, USA); anti-Osteocalcin (#DF12303, Affinity, USA); anti-Runx2 (AF5186, Affinity, USA); anti-α-SMA (AF1032, Affinity, USA); anti-Wnt3a (DF6113, Affinity, USA), anti-GSK-3β (A16868, Abcam, Cambridge, UK), anti-phospho-GSK-3β (Ser9; AP0039, Abcam, Cambridge, UK), anti-β-catenin (51,067-2-AP, Proteintech, USA), anti-phospho-β-catenin (Ser675; 4176 T, Cell Signaling Technology, USA), anti- phospho-β-catenin (Ser33/37; 4044 K, Cell Signaling Technology, USA).

### Statistical Analysis

All results are presented as mean ± standard deviation (SD) of at least three independent experiments. Statistical analysis was performed by one-way ANOVA with Bonferroni post hoc test or Student’s t-test, as stated in figure legends, using GraphPad Prism 8.0 statistical software. Values of *p* < 0.05 were considered statistically significant.

## Results

### β-GP Induces Calcification and Osteogenic Differentiation of Human VSMCs

**Fig. 1:**
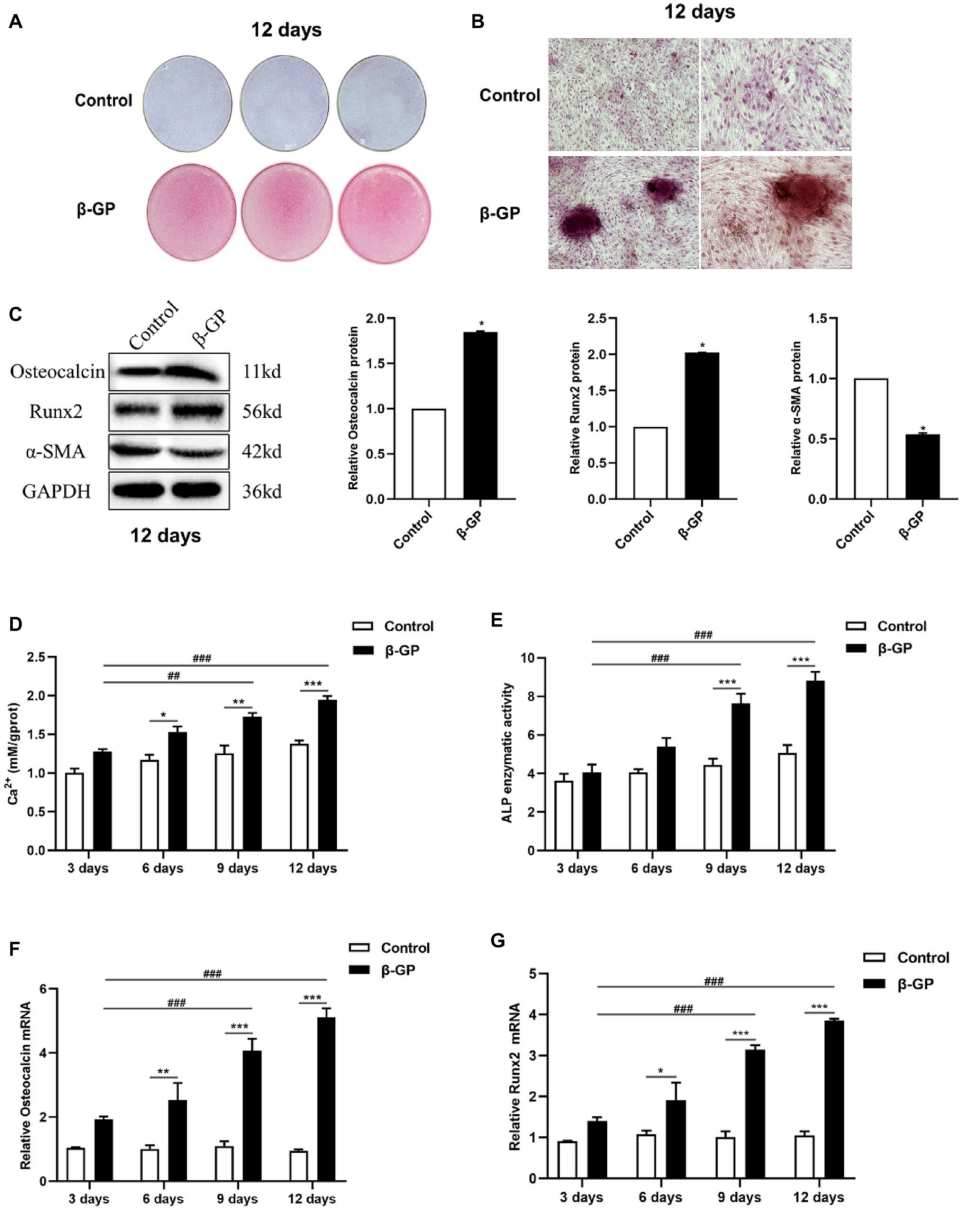
β-GP induces calcification and osteogenic differentiation of human VSMCs. Human VSMCs were treated with or without 10 mM β-GP for 12 days (*n* = 3). **a** Calcium deposition in cultured VSMCs was detected by alizarin red S staining. Scale bar = 80 mm. **b** Calcified nodules in cultured VSMCs was detected by alizarin red S staining. Scale bar = 100 µm. **c** Osteocalcin, Runx2, and α-SMA protein levels were analyzed by Western blot and quantified by densitometry. Statistical significance was assessed using t-test. All graphs show mean + SD. **d** Calcium content was measured as described in methods. **e** ALP activity was assessed by spectrophotometry. **f** Osteocalcin mRNA expression was determined by qRT-PCR. **g** Runx2 mRNA expression was determined by qRT-PCR. Statistical significance was assessed using t-test or ANOVA. All graphs show mean + SD. *P < 0.05, **P < 0.01, ***P < 0.001 vs. Control. ^**##**^*P* < 0.01, ^**###**^*P* < 0.001 vs. β-GP for 3 days

To examine whether β-GP can stimulate VSMCs calcification and osteogenic differentiation, human VSMCs were incubated with 10 mM β-GP for 12 days. We found that β-GP-treated cells presented obvious calcification, but no sign of calcification was observed in the blank controls, as observed by alizarin red S staining (Fig. 1a and b). Western blot analysis showed that β-GP increased significantly the expression of osteogenic differentiation-related genes including Runx2 and Osteocalcin in human VSMCs, whereas β-GP reduced the expression of α-SMA in human VSMCs, compared with control groups (Fig. 1c). These results indicated that β-GP promoted VSMCs calcification and osteogenic differentiation. VSMCs calcification and osteogenic differentiation were associated with the loss of contractile phenotypic proteins.

To further observe the characteristics of calcifying VSMCs, we examined the levels of calcium and gene expression of osteoblast markers in human VSMCs treated with 10 mM β-GP over a time span of 12 days (Fig. 1d, e, f, g). The results showed that β-GP-increased calcium content, as well as Runx2 and Osteocalcin mRNA levels, became detectable after culture of 6 days, whereas β-GP-enhanced ALP activity became detectable after culture of 9 days, indicating that ALP activity may be not suitable as an early indicator for predicting VSMCs calcification. Moreover, we also found that the levels of calcium, ALP activity, and Runx2 and Osteocalcin mRNA expressions in β-GP-treated cells for 9 or 12 days were significantly higher than those in β-GP-treated cells for 3 days, by comparing β-GP group at different times, suggesting that VSMCs calcification and osteogenic differentiation became more pronounced with the prolongation of β-GP treatment.

### RTEF-1 Expression is decreased in β-GP-Induced Human VSMCs

**Fig. 2:**
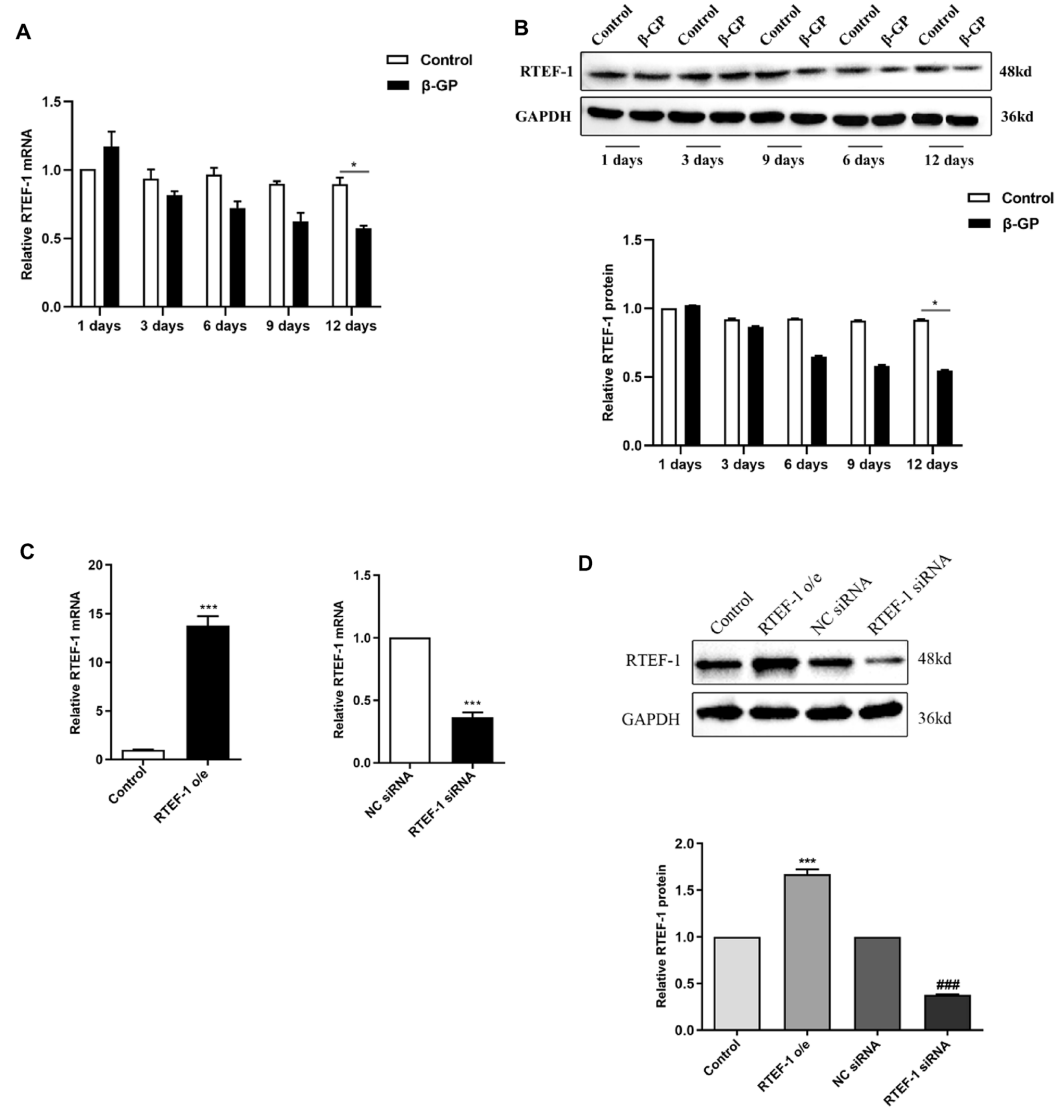
β-GP induces the downregulation of RTEF-1 expression in human VSMCs and RTEF-1 genes with different expressed backgrounds are constructed in human VSMCs. Human aortic VSMCs were treated with or without 10 mM β-GP for 12 days (**a** and **b**, *n* = 3). RTEF-1-overexpressed VSMCs, RTEF-1 siRNA VSMCs ,or control VSMCs were constructed (**c** and **d**, *n* = 3). **a** RTEF-1 mRNA expression was determined by qRT-PCR. **b** RTEF-1 protein expression was analyzed by Western blot and quantified by densitometry. **c** RTEF-1 mRNA expression was detected in control VSMCs, RTEF-1 overexpressed VSMCs, negative siRNA VSMCs, or RTEF-1 siRNA VSMCs by qRT-PCR. **d** RTEF-1 protein expression was determined in control VSMCs, RTEF-1 overexpressed VSMCs, negative siRNA VSMCs, or RTEF-1 siRNA VSMCs by Western blot, and quantified by densitometry. Statistical significance was assessed using t-test. All graphs show mean + SD. ***P < 0.001 vs. Control. ^**###**^*P* < 0.001 vs. NC siRNA

To determine the change of RTEF-1 gene expression during VSMCs calcification, human VSMCs were incubated with 10 mM β-GP over a time span of 12 days (Fig. 2a and b). Both RT-PCR and Western blot analysis displayed that β-GP significantly downregulated the expression of RTEF-1 in human VSMCs after 12 days, but no significant difference was detected in β-GP-treated VSMCs after culture of 1, 3, 6, or 9 day(s), compared with control groups. These results demonstrated that RTEF-1 was reduced in calcified VSMCs and that RTEF-1 might play a role in β-GP-induced VSMC calcification.

### RTEF-1 Ameliorates β-GP-Induced Calcification and Osteogenic Differentiation of Human VSMCs

**Fig. 3:**
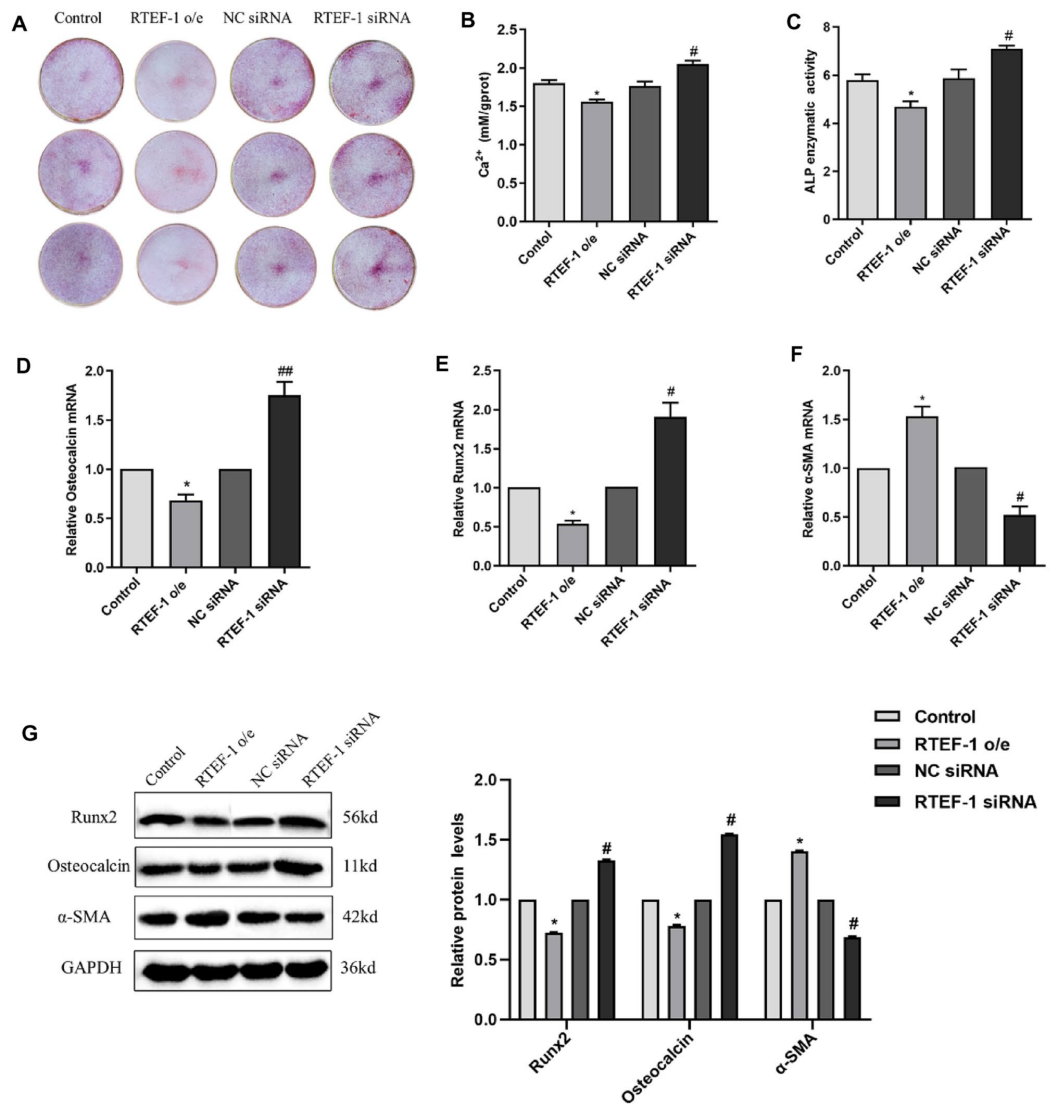
RTEF-1 prevents β-GP-induced calcification and osteogenic differentiation of human VSMCs. RTEF-1-overexpressed VSMCs, RTEF-1 siRNA VSMCs, or control VSMCs were treated in the presence of 10 mM β-GP for 9 days (*n* = 3). **a** Calcium deposition in cultured VSMCs was detected by alizarin red S staining. Scale bar = 80 mm. **b** Calcium content was measured. **c** ALP activity was assessed by spectrophotometry. **d** Osteocalcin mRNA expression was determined by qRT-PCR. **e** Runx2 mRNA expression was analyzed by qRT-PCR. **f** α-SMA mRNA expression was detected by qRT-PCR. **g** Western blot analysis of Osteocalcin, Runx2, and α-SMA protein expressions. Statistical significance was assessed using t-test. All graphs show mean + SD. *P < 0.05 vs. Control. ^**#**^*P* < 0.05 vs. NC siRNA

RTEF-1-overexpressed (o/e) and RTEF-1 siRNA VSMCs were treated with 10 mM β-GP for 9 days to investigate the effect of RTEF-1 on β-GP-induced VSMCs calcification and osteogenic differentiation. RTEF-1 expression levels in RTEF-1 o/e and siRNA VSMCs were detected by both RT-PCR and Western blot (Fig. 2c and d). As observed by alizarin red S staining (Fig. 3a), all cultured VSMCs were calcified after β-GP treatment. However, overexpression of RTEF-1 significantly reduced β-GP-induced calcification. While knockdown of RTEF-1 by small interfering RNA (siRNA) significantly increased β-GP-induced calcification. Accordingly, exposed to β-GP, the levels of calcium were decreased in RTEF-1 o/e VSMCs (1.56  ±  0.04), compared with control VSMCs (1.81  ±  0.07) (Fig. 3b). Consistently, calcium content was increased in RTEF-1 siRNA VSMCs (2.05  ±  0.07), compared with negative control VSMCs (1.76  ±  0.09), indicating the protective effect of RTEF-1 on VSMCs calcification induced by β-GP.

In addition, we explored the role of RTEF-1 in VSMCs osteogenic differentiation induced by β-GP. We found that overexpression of RTEF-1 remarkedly decreased the activity of ALP, whereas RTEF-1 siRNA increased the activity of ALP (Fig. 3c). RT-PCR revealed that the mRNA levels of Osteocalcin and Runx2 were significant reduced in RTEF-1 o/e VSMCs by 32% and by 46%, respectively, but obviously increased in RTEF-1 siRNA VSMCs (Fig. 3d and e). Accordingly, a 53% upregulation in the expression of α-SMA mRNA was detected in RTEF-1o/e VSMCs, while a 52% downregulation in the expression of α-SMA mRNA was detected in RTEF-1 siRNA VSMCs (Fig. 3f). Consistent with the mRNA expression, Western blot confirmed that overexpression of RTEF-1 reduced the protein expressions of Runx2 and Osteocalcin, but increased α-SMA protein expression (Fig. 3g). The expressions of Osteocalcin, Runx2, and α-SMA protein in RTEF-1 siRNA VSMCs showed an opposite result. Therefore, we proposed that RTEF-1 was involved in preventing β-GP-induced osteogenic differentiation of VSMCs by controlling SMC contractile phenotype.

### RTEF-1 Inhibits the Activation of the Wnt/β‐Catenin Signaling Pathway to Ameliorate β-GP‐Induced Calcification of Human VSMCs

**Fig. 4:**
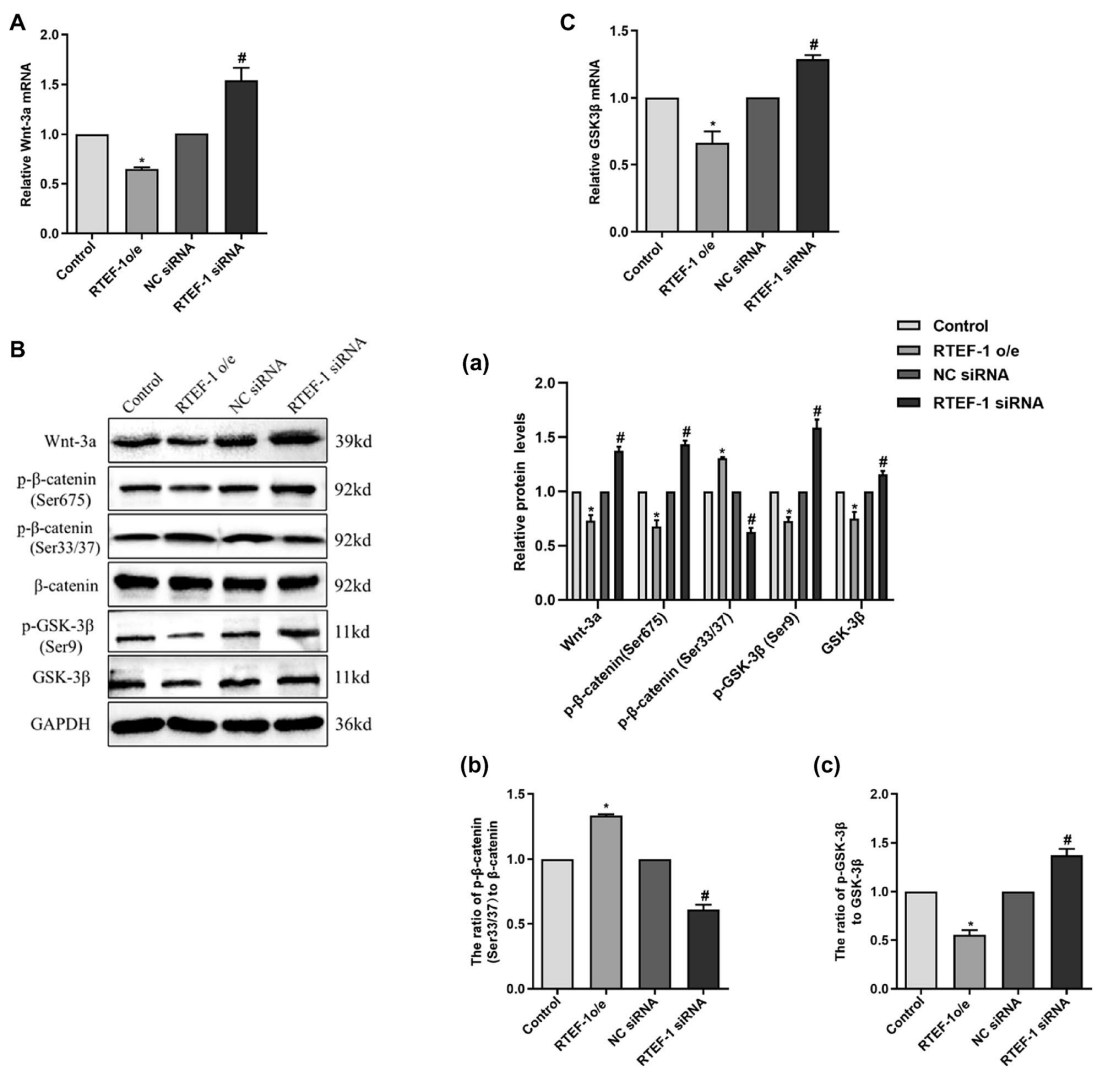
RTEF-1 inhibits β-GP-activated Wnt/β‐catenin signaling pathway in human VSMCs. RTEF-1-overexpressed VSMCs, RTEF-1-siRNA VSMCs, or control VSMCs were treated in the presence of 10 mM β-GP for 9 days (*n* = 3). **A** Wnt3a mRNA levels were analyzed by qRT-PCR. **B** Wnt3a, p-β‐catenin (Ser675), p-β‐catenin (Ser33/37), β‐catenin, p-GSK-3β (Ser9), and GSK-3β protein expression were analyzed by Western blot and quantified by densitometry. **C** GSK-3β mRNA expression was detected by qRT-PCR. **a** Western blotting analysis of Wnt3a, p-β‐catenin (Ser675), p-β‐catenin (Ser33/37), p-GSK-3β (Ser9), and GSK-3β. **b** Western blotting analysis of the ratio of p-β‐catenin (Ser33/37) to β‐catenin protein, **c** Western blotting analysis of the ratio of p-GSK-3β to GSK-3β protein. Statistical significance was assessed using t-test. All graphs show mean + SD. *P < 0.05 vs. Control. ^#^*P* < 0.05 vs. NC siRNA

RTEF-1 o/e and RTEF-1 siRNA VSMCs were treated with 10 mM β-GP for 9 days, followed by detecting the levels of Wnt3a, β-catenin, p-β-catenin (Ser675), p-β-catenin (Ser33/37), GSK-3β, and p-GSK-3β (Ser9) in cultured VSMCs (Fig. 4a, b and c). RT‐PCR and Western blot analysis showed a downregulated expression of Wnt3a in RTEF-1 o/e VSMCs, but an upregulated expression of Wnt3a in RTEF-1 siRNA VSMCs, compared with control VSMCs. Also, RTEF-1 o/e significantly decreased the protein levels of p-β-catenin (Ser675), whereas RTEF-1 siRNA increased the protein levels of p-β-catenin (Ser675). The results showed that RTEF-1 reduced the phosphorylated modification of β-catenin on Ser675 site, thereby inhibiting its transcriptional activity and the expression of bone-related genes. As shown in Fig. 4b (a) and (b), p-β-catenin (Ser33/37) protein levels and the ratio of p-β-catenin (Ser33/37) to β-catenin protein were enhanced in RTEF-1 o/e VSMCs, but reduced in RTEF-1 siRNA VSMCs. The phosphorylation of β-catenin on the Ser33/37 sites reduces its stabilization and promotes its degradation through the proteaosome pathway. Therefore, we deduced that RTEF-1 also accelerated the degradation of β-catenin in calcified VSMCs thus reducing the further expression of bone-related proteins and the formation of calcified VSMCs.

Additionally, given that the phosphorylation of β-catenin on the Ser33/37 sites can be achieved by GSK-3β, we tested the role of RTEF-1 on the expression and activity of GSK-3β in human VSMCs, as shown in Fig. 4b (a), (c), and c. We found that GSK-3β mRNA and protein expressions were reduced in RTEF-1 o/e VSMCs, whereas GSK-3β mRNA and protein expressions were increased in RTEF-1 siRNA VSMCs, when exposed to β-GP conditions, suggesting that RTEF-1 has an influence on the expressional levels of GSK-3β. As shown in Fig. 4b (a), RTEF-1 was capable to decrease the protein expression of p-GSK-3β (Ser9) in calcified VSMCs. The phosphorylation of GSK-3β on the Ser9 site was widely accepted to inhibit its own activation and the subsequent phosphorylation of β-catenin on Ser33/Ser37/Thr41 sites, thereby reducing the degradation of β-catenin in the cytoplasm. The ratio of p-GSK-3β (Ser9) to GSK-3β protein was decreased in RTEF-1 o/e VSMCs, but increased in RTEF-1 siRNA VSMCs, suggesting that RTEF-1 also promoted the activation of GSK-3β through its decreased phosphorylated modification on the Ser9 site.

**Fig. 5:**
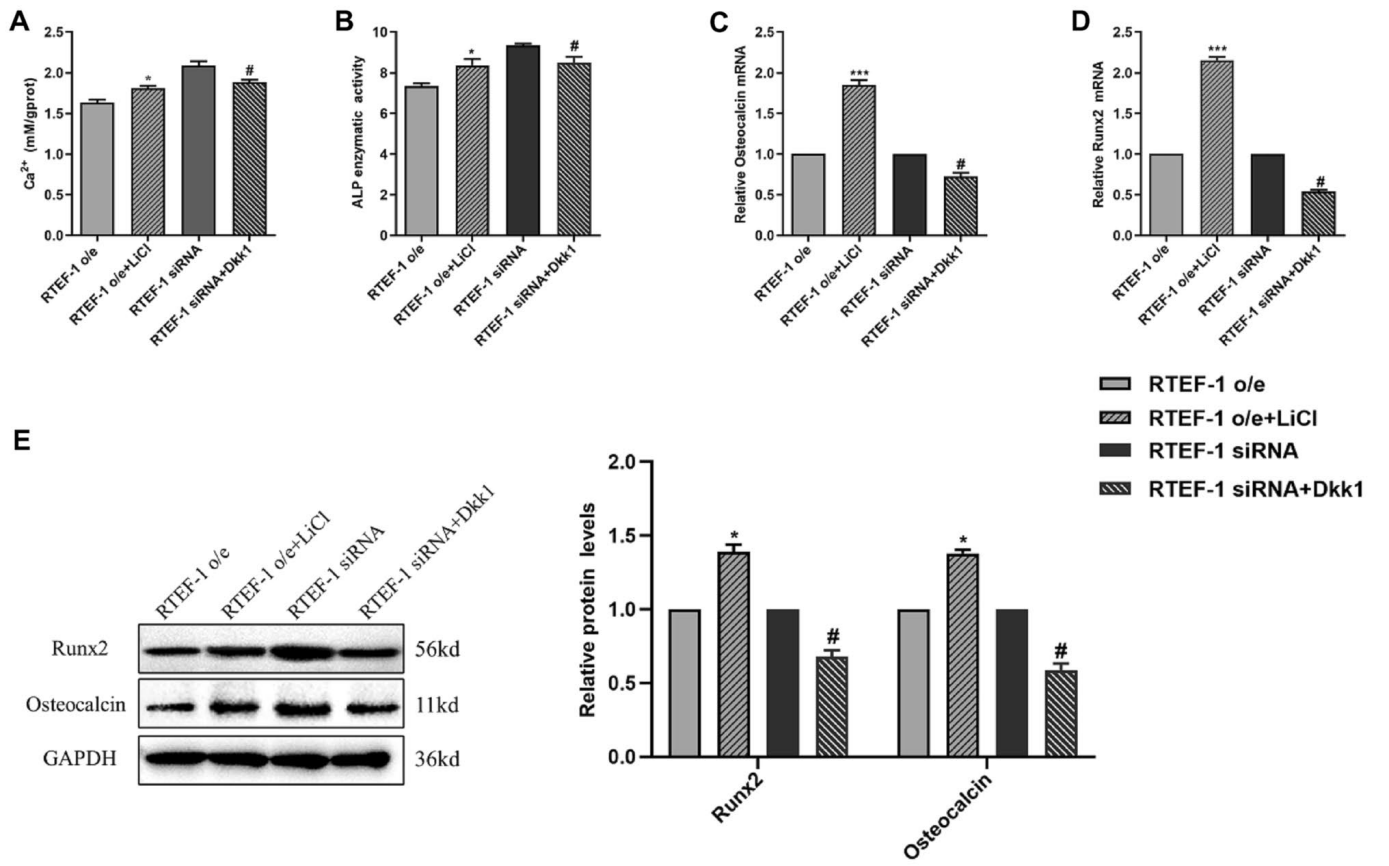
The Wnt/β‐catenin signaling pathway is involved in RTEF-1-driven anti-calcifying effect in VSMCs. RTEF-1-overexpressed VSMCs were treated in the presence of 10 mM β-GP for 9 days, with or without 5 mmol/L LiCl added for the last 3 days of this period (*n* = 3). RTEF-1 siRNA VSMCs were treated in the presence of 10 mM β-GP for 9 days, with or without 100 ng/ml Dkk1 for the last 3 days of this period (*n* = 3). **a** Calcium content was measured. **b** ALP activity was assessed by spectrophotometry. **c** Osteocalcin mRNA expression was determined by qRT-PCR. **d** Runx2 mRNA expression was analyzed by qRT-PCR. **e** Western blot analysis of Osteocalcin and Runx2 protein expressions. Statistical significance was assessed using t-test. All graphs show mean + SD. *P < 0.05, ***P < 0.001 vs. RTEF-1 o/e. ^**#**^*P* < 0.05 vs. RTEF-1 siRNA

We further determined whether the protective effect of RTEF-1 on calcification induced by β-GP-induced VSMCs is mediating through the inhibition of the Wnt/β-catenin signaling pathway. An activator or inhibitor of Wnt/β-catenin signaling pathway was used to intervene β-GP-treated RTEF-1 o/e VSMCs and RTEF-1 siRNA VSMCs, respectively, and gene expression of calcification and osteoblast markers was examined in human VSMCs. LiCl-treated RTEF-1 o/e group displayed enhanced levels of calcium and Dkk1-treated RTEF-1 siRNA group presented reduced levels of calcium (Fig. 5a). Similarly, the activity of ALP was increased in LiCl-treated RTEF-1 o/e group, whereas the activity of ALP was decreased in Dkk1-treated RTEF-1 siRNA group (Fig. 5b). RT-PCR and Western blot analysis displayed upregulated expressions of Osteocalcin and Runx2 in LiCl-treated RTEF-1 o/e, but downregulated expressions of Osteocalcin and Runx2 in Dkk1-treated RTEF-1 siRNA group (Fig. 5c, d, and e). These results suggested that LiCl reversed the protective effect of RTEF-1 overexpression on VSMCs calcification and osteogenic differentiation induced by β-GP and that Dkk1 attenuated the role of RTEF-1 deficiency in β-GP-induced VSMCs calcification. Taken together, it deduced that RTEF-1 suppressed the activation of the Wnt/β‐catenin signaling pathway to ameliorate β-GP‐induced calcification of human VSMCs.

## Discussion

Our results reveal novel aspects for the function of the multifunctional transcription factor RTEF-1 in VC. We revealed that RTEF-1 was present in low levels in calcified VSMCs, this was accompanied by a decrease in α-SMA expression and increased levels of osteogenic markers including ALP, osteocalcin, and Runx2. In addition, we found that RTEF-1 ameliorated β-GP-induced VSMC calcification and osteogenic differentiation, the underlying mechanism of which was associated with negatively regulating Wnt/β-catenin signaling pathway. VSMCs calcification and osteogenic differentiation were associated with the loss of contractile phenotypic proteins.

To our knowledge, to date, there are no reports showing a direct association between RTEF-1 and VC. Previous studies showed that RTEF-1 as a downstream effector of the Hippo pathway has important roles in cancer, including epithelial–mesenchymal transition (EMT), metastasis, cancer stem cell dynamics, and chemotherapeutic drug resistance, suggesting that RTEF-1 may be a promising prognostic biomarker in cancer [9]. Apart from its role in cancers, RTEF-1 is also a critical regulator of cardiac and smooth muscle-specific genes during cardiovascular development and cardiac disorders including cardiac hypertrophy [12]. This study found that RTEF-1 expression was reduced in calcified VSMCs, which may be associated with the loss of contractile phenotypic proteins or the activation of Wnt/β-catenin signaling pathway. VSMCs are of mesenchymal origin and, under stress, can differentiate into different mesenchymal-derived cell types, such as osteoblasts and chondrocytes, leading to calcification [3]. Also, VSMCs can trans-differentiate into proliferative phenotype, characterized by increased proliferation and migration of the cells toward the intima as well as the enhanced synthesis of extracellular matrix proteins, proteases, and cytokines, thus contributing to the healing process by formation of a neointima [21]. The main characteristic of these phenotypic changes is the loss of SM-specific genes. RTEF-1 was showed to promote the expression of SM-specific gene, such as α-SMA, which is not similar to the function of TEAD1, another member of the TEAD family. TEAD1 was reported to represses the expression of SM-specific gene, such as SM MHC, myosin light chain kinase, Hic-5, SM-actin, calponin, and SM22, but increase the expression of the proliferative marker, by abolishing Myocardin function [22]. Each member of the TEAD family shows tissue-specific expression, indicating that they have both common functions and distinct functions [9]. Our study showed that RTEF-1 downregulated the expression of bone-related proteins and upregulated the expression of contractile protein in VSMCs, suggesting the protective effect of RTEF-1 on VC by controlling trans-differentiation of VSMCs from a contractile to osteogenic phenotype.

Activation of Wnt/β-catenin signaling by pro-calcifying factors is essential for osteoblast differentiation and phenotypic characterization of VSMCs through governing the expression of bone-related proteins [17]. Both canonical and non-canonical Wnt proteins, such as Wnt1, Wnt3a, Wnt8, Wnt8b, Wnt4, and Wnt5a, were shown to be upregulated in calcified VSMCs and as an upstream activator to activate cell surface receptor-mediated signal transduction pathways to regulate target gene expression [15]. RTEF-1 was demonstrated to decrease the expression of Wnt3a, which is a canonical Wnt protein known to promote the osteogenic differentiation of VSMCs by enhancing the transcriptional activity of β-catenin, so active β-catenin would be reduced in RTEF-1 o/e VSMCs. Two forms of active β-catenin, dephosphorylated on Ser37/Thr41 and phosphorylated on Ser675 site, were shown to be upregulated by high phosphate in VSMCs [17]. RTEF-1 reduced the expression of p-β-catenin (Ser675), thereby inhibiting its transcriptional activity and the expression of bone-related genes. In addition, GSK-3β-mediated phosphorylation of β-catenin on the Ser33/Ser37 sites were shown to increase in RTEF-1 o/e VSMCs, but decreased in RTEF-1 siRNA VSMCs, compared with control VSMCs. This result showed that RTEF-1 also accelerated the degradation of β-catenin in calcified VSMCs, thus reducing the further expression of bone-related proteins and the formation of calcified VSMCs. The degradation of β-catenin can be achieved by the activation of GSK-3β, and we also tested the role of RTEF-1 on the expression and activity of GSK-3β in human VSMCs. We found that RTEF-1 was capable to decrease the expression of GSK-3β and reduce the levels of p-GSK-3β (Ser9) protein, as well as increasing the ratio of p-GSK-3β (Ser9) to GSK-3β protein in VSMCs. The phosphorylation of GSK-3β on the Ser9 site was widely accepted to inhibit its own activation and the subsequent phosphorylation of β-catenin on Ser33/Ser37/Thr41 sites, thereby reducing the degradation of β-catenin in the cytoplasm. RTEF-1 deceased the levels of inactive GSK-3β, which would enhance the degradation of β-catenin in the cytoplasm, eventually leading to the reduction of bone-related proteins expression and calcified VSMCs, being in accordance with the role of RTEF-1 on the phosphorylation of β-catenin (Ser33/Ser37). Other studies have reported RTEF-1 participated in post-translational modifications, crosstalk between cancer-related signaling pathways, and cancer-causing mutations [9]. This study showed that RTEF-1 not only changed the expressions of Wnt3a, but also affected the phosphorylated modifications of β-catenin and GSK-3β to change their function, thereby regulating the Wnt/β-catenin signaling. Additionally, YAP/TAZ has been shown to inhibit the osteoblast differentiation in bone development by promoting the proteasomal degradation of β-catenin and antagonizing release of β-catenin from the destruction complex [20]. Cooperation of RTEF-1, as a downstream effector of the Hippo-YAP/TAZ pathway, with transcription factors is a common cellular phenomenon. RTEF-1 was reported to be regulated by PI3K/AKT/β-catenin signaling, which activated the transcription of MMP9, a key molecule that promotes gastric cancer cell proliferation and metastasis [23]. Furthermore, specific activator or inhibitor of Wnt/β-catenin signaling used in RTEF-1 overexpressed or siRNA VSMCs, respectively, has further suggested that Wnt/β-catenin signaling was involved in the anti-calcifying effect of RTEF-1. Dkk1, a common Wnt antagonist, was shown to be elevated in patients with CKD-associated bone and mineral disorder and neutralization of Dkk1 by a monoclonal antibody was shown to prevent osteogenic trans-differentiation of VSMCs, VC, and renal osteodystrophy [24]. This study found that Dkk1 alleviated the role of RTEF-1 deficiency in high phosphate-induced VSMC calcification. Accordingly, treatment with LiCl, activator of Wnt/β-catenin signaling, reversed the protective effects of RTEF-1 on high phosphate-induced calcification and osteogenic differentiation of human VSMCs. In conclusion, our results demonstrate for the first time that RTEF-1 ameliorates β-GP-induced VSMCs calcification and osteogenic differentiation through regulation of Wnt/β-catenin signaling and highlights the critical role of RTEF-1 in VC. Further understanding of the role of RTEF-1 using in vivo model of VC would provide a novel insight into the therapeutic target.

## Data Availability

All data used or analyzed during this study are available from the corresponding author on reasonable request.

## Code Availability

All codes used or analyzed during this study are available from the corresponding author on reasonable request.
